# P-75. Comparison of clinical characteristics and Outcome of Brucella Vertebral Osteomyelitis and Pyogenic Vertebral Osteomyelitis: A 5 year retrospective Study

**DOI:** 10.1093/ofid/ofaf695.304

**Published:** 2026-01-11

**Authors:** Ajithkumar Ittaman, Junais koleri, Moideenkutty Gurukkal, Muna Maslamani, A S H A ALEX

**Affiliations:** HAMAD MEDICAL CORPORATION, Thrissur, Kerala, India; HAMAD MEDICAL CORPORATION, Thrissur, Kerala, India; HAMAD MEDICAL CORPORATION, Thrissur, Kerala, India; HAMAD MEDICAL CORPORATION, Thrissur, Kerala, India; HAMAD MEDICAL CORPORATION, Thrissur, Kerala, India

## Abstract

**Background:**

Vertebral osteomyelitis (VO) is a serious infection with varying clinical courses depending on the underlying etiology. This study compares the clinical profile, radiological features, and outcomes of Brucella vertebral osteomyelitis (BVO) and Pyogenic vertebral osteomyelitis (PVO).

Outcome and subgroup anlysis

**Figure:**
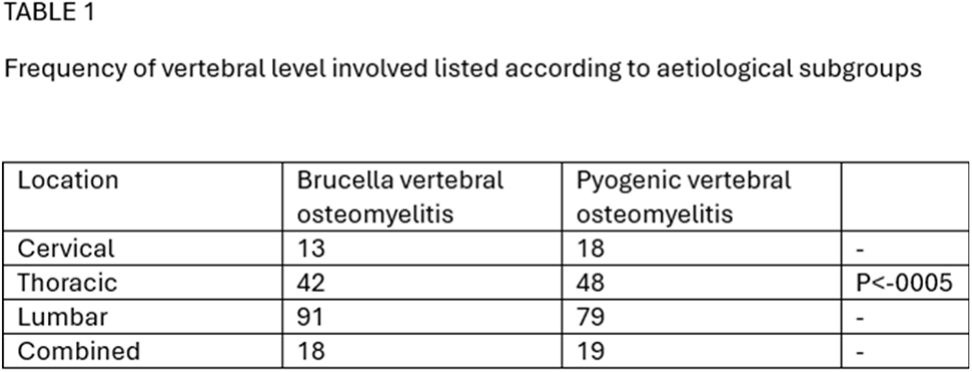

**Methods:**

A comparative study was conducted involving 328 patients—164 with BVO were compared with 164 with PVO, randomized and matched based on age, gender and presence of diabetes. Demographics, vertebral involvement, clinical presentation, treatment modalities, and outcomes were evaluated. Functional recovery was assessed using EQ-5D scores at 1 and 2 years.

**Figure:**
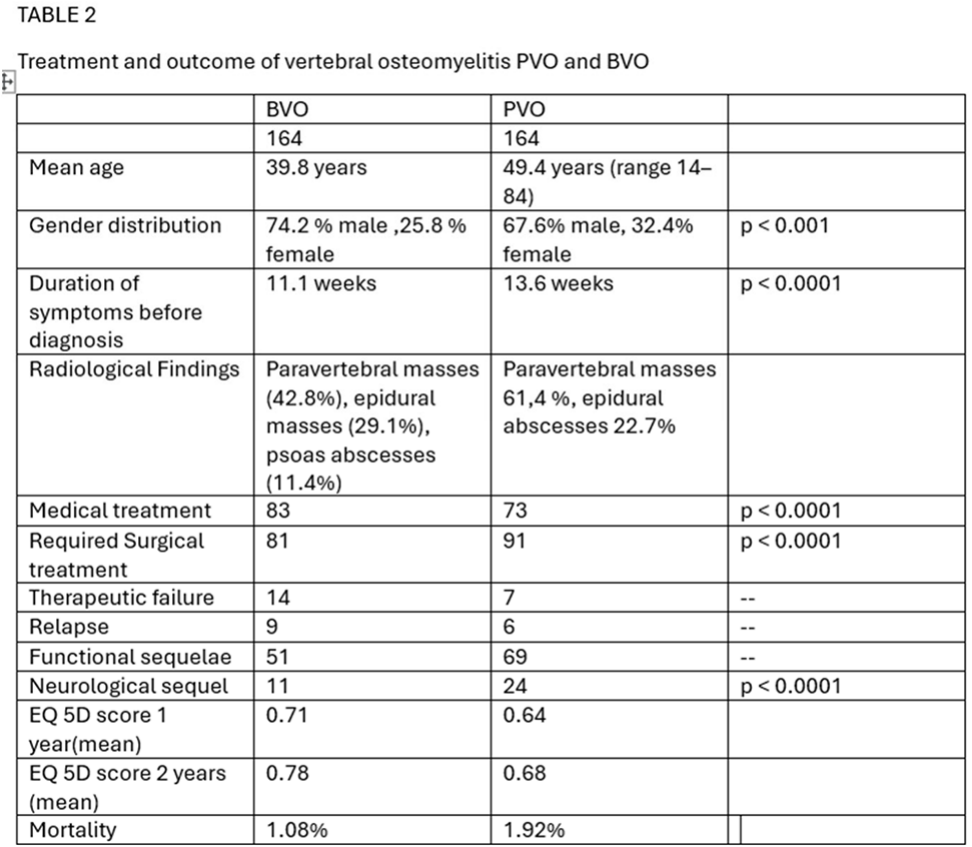

- Results: Age of onset was earlier in BVO (mean 39.8 years) compared to PVO (mean 49.4 years).
- Gender distribution showed male predominance in both groups (74.2% BVO vs. 67.6% PVO, *p* < 0.001).
- Duration of symptoms before diagnosis was shorter in BVO (11.1 weeks vs. 13.6 weeks, *p* < 0.0001).
- Radiological findings differed between groups, with paravertebral masses more common in PVO (61.4%) than in BVO (42.8%). BVO cases showed higher incidences of epidural and psoas abscesses.
- Vertebral involvement: Lumbar spine was most frequently affected in both groups. Thoracic vertebrae involvement was significantly higher in PVO (*p* < 0.0005).
- Treatment: Medical therapy alone was more often sufficient in BVO (83 vs. 73, *p* < 0.0001), whereas PVO more frequently required surgical intervention (91 vs. 81, *p* < 0.0001).
- Outcomes: Neurological sequelae were significantly lower in BVO (11 vs. 24, *p* < 0.0001). Functional recovery was better in BVO, with higher EQ-5D scores at 1 year (0.71 vs. 0.64) and 2 years (0.78 vs. 0.68).
- Rates of therapeutic failure, relapse, and functional sequelae were comparable between groups.

**Conclusion:**

BVO patients had an earlier onset, requiring lesser surgical intervention fewer neurological complications and had lesser requirement of surgical intervention. BVO had better functional outcomes and higher EQ-5D scores at 1 and 2 years compared to PVO. This study highlights the prognostic implications of underlying aetiology in vertebral osteomyelitis.

**Disclosures:**

All Authors: No reported disclosures

